# 2-(4-Bromo­phen­yl)-*N*-(pyrazin-2-yl)acetamide

**DOI:** 10.1107/S1600536813012531

**Published:** 2013-05-15

**Authors:** Prakash S. Nayak, B. Narayana, Jerry P. Jasinski, H. S. Yathirajan, B. K. Sarojini

**Affiliations:** a Department of Studies in Chemistry, Mangalore University, Mangalagangotri 574 199, India; b Department of Chemistry, Keene State College, 229 Main Street, Keene, NH 03435-2001, USA; c Department of Studies in Chemistry, University of Mysore, Manasagangotri, Mysore 570 006, India; d Department of Chemistry, P.A. College of Engineering, Nadupadavu, Mangalore 574 153, India

## Abstract

In the title compound, C_12_H_10_BrN_3_O, the dihedral angle between the mean planes of the 4-bromo­phenyl and pyrazin-2-yl rings is 54.6 (3)°. An intra­molecular C—H⋯O hydrogen bond generates an *S*(6) graph-set motif. In the crystal, weak N—H⋯N hydrogen bonds link the mol­ecules into chains along [100]. The chains are linked *via* C—H⋯N and C—H⋯O hydrogen bonds, forming two-dimensional networks lying parallel to the *ab* plane.

## Related literature

For the structural similarity of *N*-substituted 2-aryl­acetamides to the lateral chain of natural benzyl­penicillin, see: Mijin & Marinkovic (2006 ▶); Mijin *et al.* (2008 ▶). For the coordination abilities of amides, see: Wu *et al.* (2008 ▶, 2010 ▶). For related structures, see: Fun *et al.* (2012*a* ▶,*b* ▶,*c* ▶,*d* ▶). For bond-length data, see: Allen *et al.* (1987 ▶).
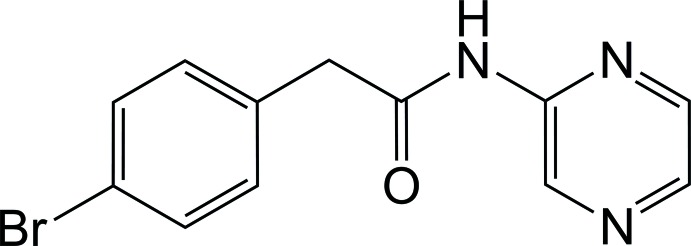


## Experimental

### 

#### Crystal data

C_12_H_10_BrN_3_O
*M* *_r_* = 292.14
Orthorhombic, 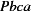

*a* = 10.6804 (4) Å
*b* = 7.5196 (3) Å
*c* = 29.1355 (10) Å
*V* = 2339.94 (14) Å^3^
*Z* = 8
Cu *K*α radiation
μ = 4.69 mm^−1^
*T* = 173 K
0.16 × 0.08 × 0.06 mm

#### Data collection

Agilent Xcalibur Eos Gemini diffractometer
Absorption correction: multi-scan (*CrysAlis PRO* and *CrysAlis RED*; Agilent, 2012 ▶) *T*_min_ = 0.388, *T*_max_ = 1.000
13941 measured reflections
2317 independent reflections
2036 reflections with *I* > 2σ(*I*)
*R*_int_ = 0.036

#### Refinement

*R*[*F* ^2^ > 2σ(*F* ^2^)] = 0.035
*wR*(*F* ^2^) = 0.095
*S* = 1.03
2317 reflections
158 parameters
H atoms treated by a mixture of independent and constrained refinement
Δρ_max_ = 0.75 e Å^−3^
Δρ_min_ = −0.81 e Å^−3^

### 

Data collection: *CrysAlis PRO* (Agilent, 2012 ▶); cell refinement: *CrysAlis PRO*; data reduction: *CrysAlis PRO*; program(s) used to solve structure: *SHELXS97* (Sheldrick, 2008 ▶); program(s) used to refine structure: *SHELXL2012* (Sheldrick, 2008 ▶); molecular graphics: *OLEX2* (Dolomanov *et al.*, 2009 ▶); software used to prepare material for publication: *OLEX2*.

## Supplementary Material

Crystal structure: contains datablock(s) global, I. DOI: 10.1107/S1600536813012531/bt6905sup1.cif

Structure factors: contains datablock(s) I. DOI: 10.1107/S1600536813012531/bt6905Isup2.hkl

Supplementary material file. DOI: 10.1107/S1600536813012531/bt6905Isup3.cml

Additional supplementary materials:  crystallographic information; 3D view; checkCIF report

## Figures and Tables

**Table 1:** Hydrogen-bond geometry (Å, °)

*D*—H⋯*A*	*D*—H	H⋯*A*	*D*⋯*A*	*D*—H⋯*A*
N1—H1⋯N3^i^	0.78 (3)	2.28 (3)	3.059 (3)	178 (3)
C3—H3⋯O1	0.93	2.24	2.848 (3)	123
C3—H3⋯N2^ii^	0.93	2.47	3.255 (3)	142
C6—H6*A*⋯O1^iii^	0.97	2.53	3.424 (4)	154

Symmetry codes: (i) 
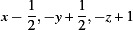
; (ii) 
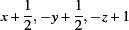
; (iii) 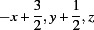.

**Fig. 1.**
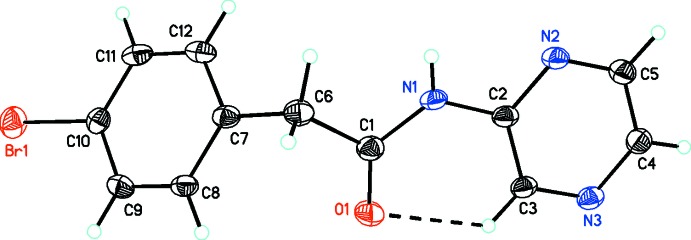
Molecular structure of the title compound showing the atom labeling scheme and 30% probability displacement ellipsoids. Dashed line indicates an intramolecular C3—H2···O1 hydrogen bond in an S(6) graph-set motif.

**Fig. 2.**
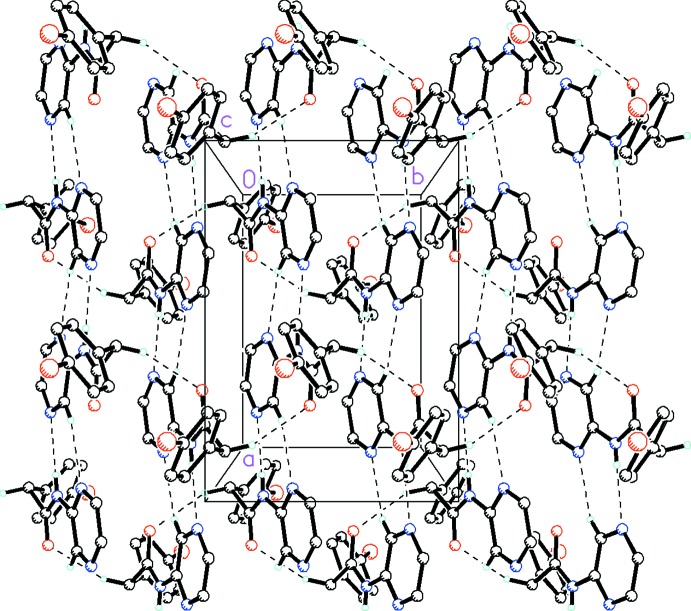
Packing diagram of the title compound viewed along the c axis. Dashed lines indicate N—H···N hydrogen bonds and weak C—H···N and C—H···O intermolecular interactions linking the molecules into chains along [100] forming 2-D networks lying parallel to the ab plane.

**Fig. 3.**
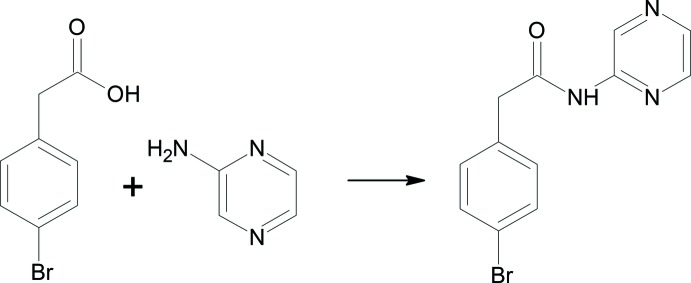
Reaction scheme.

## supplementary crystallographic information

### Comment

N-Substituted 2-arylacetamides are very interesting compounds because of their structural similarity to the lateral chain of natural benzylpenicillin (Mijin *et al.*, 2006, 2008). Amides are also used as ligands due to their excellent coordination abilities (Wu *et al.*, 2008, 2010). Crystal structures of some acetamide derivatives viz., N-(3,4-difluoro phenyl)-2,2-diphenylacetamide, 2-(4-bromophenyl)-N-(5-methylpyridin-2-yl)acetamide, N-(4-bromophenyl)-2-(4-chlorophenyl)acetamide, 2-(4-bromophenyl)-N-(3-chloro-4-fluorophenyl)acetamide, (Fun *et al.*, 2012*a*,*b*,*c*,*d*) have been reported. In view of the importance of amides, we report herein the crystal structure of the title compound, C_12_H_10_BrN_3_O, (I).

In (I) the dihedral angle between the mean planes of the 4-Bromophenyl and pyrazine rings is 54.6 (3)° (Fig. 1). An intramolecular C—H···O hydrogen bond generates an S(6) graph-set motif. Bond lengths are in normal ranges (Allen *et al.*, 1987). In the crystal, weak N—H···N hydrogen bonds link the molecules into chains along [100]. The chains are linked via weak C—H···N and C—H···O intermolecular interactions, forming two-dimensional networks lying parallel to the ab plane.

### Experimental

4-Bromophenylacetic acid (0.213 g, 1 mmol), 2-aminopyrazine (0.095 g, 1 mmol) and 1-ethyl-3-(3-dimethylaminopropyl)-carbodiimide hydrochloride (1.0 g, 0.01 mol) were dissolved in dichloromethane (20 mL) (Fig. 3). The mixture was stirred in presence of triethylamine at 273 K for about 3 h. The contents were poured into 100 ml of ice-cold aqueous hydrochloric acid with stirring, which was extracted thrice with dichloromethane. The organic layer was washed with saturated NaHCO_3_ solution and brine solution, dried and concentrated under reduced pressure to give the title compound (I). Single crystals were grown from methylene chloride by the slow evaporation method (M.P.: 433–435 K).

### Refinement

All of the H atoms were placed in their calculated positions and then refined using the riding model with Atom—H lengths of 0.93Å (CH) or 0.97Å (CH_2_). Isotropic displacement parameters for these atoms were set to 1.2 (CH, CH_2_) times *U*_eq_ of the parent atom.

### Crystal data

**Table:**

C_12_H_10_BrN_3_O	*D*_x_ = 1.659 Mg m^−^^3^
*M**_r_* = 292.14	Cu *K*α radiation, λ = 1.54184 Å
Orthorhombic, *P**b**c**a*	Cell parameters from 5193 reflections
*a* = 10.6804 (4) Å	θ = 4.1–72.4°
*b* = 7.5196 (3) Å	µ = 4.69 mm^−^^1^
*c* = 29.1355 (10) Å	*T* = 173 K
*V* = 2339.94 (14) Å^3^	Chunk, colorless
*Z* = 8	0.16 × 0.08 × 0.06 mm
*F*(000) = 1168	

### Data collection

**Table:**

Agilent Xcalibur Eos Gemini diffractometer	2036 reflections with *I* > 2σ(*I*)
Detector resolution: 16.1500 pixels mm^-1^	*R*_int_ = 0.036
ω scans	θ_max_ = 72.6°, θ_min_ = 5.1°
Absorption correction: multi-scan (*CrysAlis PRO* and *CrysAlis RED*; Agilent, 2012)	*h* = −7→13
*T*_min_ = 0.388, *T*_max_ = 1.000	*k* = −9→9
13941 measured reflections	*l* = −35→33
2317 independent reflections	

### Refinement

**Table:**

Refinement on *F*^2^	0 restraints
Least-squares matrix: full	H atoms treated by a mixture of independent and constrained refinement
*R*[*F*^2^ > 2σ(*F*^2^)] = 0.035	*w* = 1/[σ^2^(*F*_o_^2^) + (0.0494*P*)^2^ + 2.0499*P*] where *P* = (*F*_o_^2^ + 2*F*_c_^2^)/3
*wR*(*F*^2^) = 0.095	(Δ/σ)_max_ = 0.001
*S* = 1.03	Δρ_max_ = 0.75 e Å^−^^3^
2317 reflections	Δρ_min_ = −0.81 e Å^−^^3^
158 parameters	

### Special details

**Table:**

Geometry. All esds (except the esd in the dihedral angle between two l.s. planes) are estimated using the full covariance matrix. The cell esds are taken into account individually in the estimation of esds in distances, angles and torsion angles; correlations between esds in cell parameters are only used when they are defined by crystal symmetry. An approximate (isotropic) treatment of cell esds is used for estimating esds involving l.s. planes.

### Fractional atomic coordinates and isotropic or equivalent isotropic displacement parameters (Å^2^)

**Table:**

	*x*	*y*	*z*	*U*_iso_*/*U*_eq_	
Br1	0.64022 (3)	0.29843 (4)	0.81514 (2)	0.04987 (14)	
O1	0.77445 (16)	0.3961 (3)	0.58151 (7)	0.0473 (5)	
N1	0.5962 (2)	0.3332 (3)	0.54173 (7)	0.0339 (5)	
H1	0.524 (3)	0.340 (4)	0.5413 (10)	0.033 (8)*	
N2	0.55700 (18)	0.1871 (3)	0.47422 (8)	0.0357 (5)	
N3	0.81075 (18)	0.1345 (3)	0.45762 (7)	0.0351 (5)	
C1	0.6614 (2)	0.4063 (4)	0.57735 (9)	0.0346 (5)	
C2	0.6436 (2)	0.2438 (4)	0.50359 (9)	0.0315 (5)	
C3	0.7716 (2)	0.2175 (3)	0.49533 (9)	0.0332 (5)	
H3	0.8300	0.2586	0.5165	0.040*	
C4	0.7231 (2)	0.0759 (3)	0.42843 (9)	0.0367 (6)	
H4	0.7475	0.0161	0.4019	0.044*	
C5	0.5977 (2)	0.1023 (4)	0.43686 (9)	0.0378 (6)	
H5	0.5395	0.0597	0.4158	0.045*	
C6	0.5806 (2)	0.5067 (4)	0.61136 (9)	0.0374 (6)	
H6A	0.5997	0.6326	0.6093	0.045*	
H6B	0.4933	0.4910	0.6031	0.045*	
C7	0.5995 (2)	0.4464 (3)	0.66018 (9)	0.0315 (5)	
C8	0.7040 (2)	0.5003 (3)	0.68536 (8)	0.0326 (5)	
H8	0.7653	0.5691	0.6713	0.039*	
C9	0.7177 (2)	0.4531 (3)	0.73080 (9)	0.0341 (5)	
H9	0.7876	0.4897	0.7473	0.041*	
C10	0.6263 (2)	0.3510 (3)	0.75151 (9)	0.0323 (5)	
C11	0.5237 (2)	0.2907 (3)	0.72733 (10)	0.0384 (6)	
H11	0.4639	0.2191	0.7414	0.046*	
C12	0.5116 (2)	0.3387 (4)	0.68182 (10)	0.0389 (6)	
H12	0.4430	0.2980	0.6652	0.047*	

### Atomic displacement parameters (Å^2^)

**Table:**

	*U*^11^	*U*^22^	*U*^33^	*U*^12^	*U*^13^	*U*^23^
Br1	0.0638 (2)	0.0486 (2)	0.0372 (2)	−0.00515 (14)	0.00523 (12)	0.00487 (12)
O1	0.0237 (9)	0.0708 (14)	0.0474 (11)	0.0046 (9)	−0.0071 (7)	−0.0090 (10)
N1	0.0180 (10)	0.0469 (13)	0.0367 (11)	0.0001 (9)	0.0016 (8)	0.0025 (9)
N2	0.0227 (10)	0.0453 (12)	0.0390 (12)	0.0003 (9)	−0.0018 (8)	0.0025 (9)
N3	0.0256 (10)	0.0398 (12)	0.0398 (11)	0.0027 (9)	0.0021 (8)	0.0073 (9)
C1	0.0286 (12)	0.0399 (14)	0.0355 (13)	0.0008 (10)	−0.0016 (10)	0.0050 (11)
C2	0.0225 (11)	0.0376 (12)	0.0344 (13)	−0.0009 (9)	−0.0017 (9)	0.0088 (10)
C3	0.0229 (11)	0.0410 (14)	0.0356 (12)	−0.0003 (10)	0.0008 (9)	0.0081 (10)
C4	0.0336 (13)	0.0408 (14)	0.0357 (13)	0.0025 (11)	0.0005 (10)	0.0038 (11)
C5	0.0314 (12)	0.0438 (14)	0.0382 (14)	−0.0006 (11)	−0.0045 (10)	0.0026 (11)
C6	0.0274 (12)	0.0422 (14)	0.0428 (14)	0.0065 (11)	−0.0032 (10)	0.0007 (12)
C7	0.0230 (11)	0.0306 (12)	0.0409 (13)	0.0042 (9)	−0.0002 (9)	−0.0021 (10)
C8	0.0258 (11)	0.0306 (12)	0.0413 (13)	−0.0057 (10)	0.0028 (9)	−0.0013 (10)
C9	0.0319 (12)	0.0290 (12)	0.0414 (13)	−0.0045 (10)	−0.0040 (10)	−0.0023 (10)
C10	0.0362 (13)	0.0278 (11)	0.0330 (12)	0.0035 (9)	0.0044 (10)	0.0000 (10)
C11	0.0261 (12)	0.0335 (13)	0.0556 (16)	−0.0048 (10)	0.0068 (11)	0.0036 (11)
C12	0.0246 (12)	0.0392 (14)	0.0529 (16)	−0.0038 (10)	−0.0053 (10)	−0.0013 (12)

### Geometric parameters (Å, º)

**Table:**

Br1—C10	1.901 (3)	C5—H5	0.9300
O1—C1	1.216 (3)	C6—C7	1.507 (4)
N1—C1	1.365 (3)	C6—H6A	0.9700
N1—C2	1.394 (3)	C6—H6B	0.9700
N1—H1	0.78 (3)	C7—C12	1.391 (4)
N2—C2	1.330 (3)	C7—C8	1.396 (3)
N2—C5	1.334 (3)	C8—C9	1.378 (4)
N3—C3	1.331 (3)	C8—H8	0.9300
N3—C4	1.339 (3)	C9—C10	1.381 (3)
C1—C6	1.516 (4)	C9—H9	0.9300
C2—C3	1.402 (3)	C10—C11	1.379 (4)
C3—H3	0.9300	C11—C12	1.380 (4)
C4—C5	1.376 (4)	C11—H11	0.9300
C4—H4	0.9300	C12—H12	0.9300
			
C1—N1—C2	128.0 (2)	C1—C6—H6A	109.0
C1—N1—H1	120 (2)	C7—C6—H6B	109.0
C2—N1—H1	113 (2)	C1—C6—H6B	109.0
C2—N2—C5	116.8 (2)	H6A—C6—H6B	107.8
C3—N3—C4	117.3 (2)	C12—C7—C8	118.1 (2)
O1—C1—N1	123.8 (3)	C12—C7—C6	120.9 (2)
O1—C1—C6	122.1 (2)	C8—C7—C6	121.1 (2)
N1—C1—C6	114.0 (2)	C9—C8—C7	121.0 (2)
N2—C2—N1	114.5 (2)	C9—C8—H8	119.5
N2—C2—C3	121.5 (2)	C7—C8—H8	119.5
N1—C2—C3	124.0 (2)	C8—C9—C10	119.2 (2)
N3—C3—C2	120.9 (2)	C8—C9—H9	120.4
N3—C3—H3	119.5	C10—C9—H9	120.4
C2—C3—H3	119.5	C11—C10—C9	121.4 (2)
N3—C4—C5	121.3 (2)	C11—C10—Br1	119.5 (2)
N3—C4—H4	119.4	C9—C10—Br1	119.10 (19)
C5—C4—H4	119.4	C10—C11—C12	118.6 (2)
N2—C5—C4	122.1 (2)	C10—C11—H11	120.7
N2—C5—H5	118.9	C12—C11—H11	120.7
C4—C5—H5	118.9	C11—C12—C7	121.6 (2)
C7—C6—C1	113.0 (2)	C11—C12—H12	119.2
C7—C6—H6A	109.0	C7—C12—H12	119.2
			
C2—N1—C1—O1	−3.2 (4)	N1—C1—C6—C7	127.0 (2)
C2—N1—C1—C6	175.4 (2)	C1—C6—C7—C12	−103.9 (3)
C5—N2—C2—N1	179.3 (2)	C1—C6—C7—C8	77.7 (3)
C5—N2—C2—C3	0.8 (4)	C12—C7—C8—C9	−2.1 (4)
C1—N1—C2—N2	−179.9 (2)	C6—C7—C8—C9	176.4 (2)
C1—N1—C2—C3	−1.4 (4)	C7—C8—C9—C10	0.0 (4)
C4—N3—C3—C2	−0.8 (4)	C8—C9—C10—C11	2.0 (4)
N2—C2—C3—N3	0.0 (4)	C8—C9—C10—Br1	−176.14 (19)
N1—C2—C3—N3	−178.4 (2)	C9—C10—C11—C12	−1.8 (4)
C3—N3—C4—C5	0.8 (4)	Br1—C10—C11—C12	176.3 (2)
C2—N2—C5—C4	−0.8 (4)	C10—C11—C12—C7	−0.4 (4)
N3—C4—C5—N2	0.0 (4)	C8—C7—C12—C11	2.3 (4)
O1—C1—C6—C7	−54.4 (4)	C6—C7—C12—C11	−176.2 (2)

### Hydrogen-bond geometry (Å, º)

**Table:**

*D*—H···*A*	*D*—H	H···*A*	*D*···*A*	*D*—H···*A*
N1—H1···N3^i^	0.78 (3)	2.28 (3)	3.059 (3)	178 (3)
C3—H3···O1	0.93	2.24	2.848 (3)	123
C3—H3···N2^ii^	0.93	2.47	3.255 (3)	142
C6—H6*A*···O1^iii^	0.97	2.53	3.424 (4)	154

Symmetry codes: (i) *x*−1/2, −*y*+1/2, −*z*+1; (ii) *x*+1/2, −*y*+1/2, −*z*+1; (iii) −*x*+3/2, *y*+1/2, *z*.
